# Reference values and sex differences in absolute and relative kidney size. A Swiss autopsy study

**DOI:** 10.1186/s12882-020-01946-y

**Published:** 2020-07-20

**Authors:** Sabrina Addidou Kalucki, Christelle Lardi, Jonas Garessus, Alain Kfoury, Silke Grabherr, Michel Burnier, Menno Pruijm

**Affiliations:** 1 Service of Nephrology and Hypertension, Lausanne University Hospital and University of Lausanne, Rue du Bugnon 17, 1011 Lausanne, Switzerland; 2 University Center of Legal Medicine Lausanne-Geneva, Rue Michel-Servet 1, Geneva University Hospital, Geneva, Switzerland; 3 UUniversity Center of Legal Medicine Lausanne-Geneva, Chemin de la Vulliette 4, University Hospital of Lausanne and University of Lausanne, Lausanne, Switzerland

**Keywords:** Nephron mass, Autopsy, Gender, Sex, Chronic kidney disease, Reference values

## Abstract

**Background:**

Men have larger kidneys than women, but it is unclear whether gender remains an independent predictor of kidney size (expressed as weight or length) after correction for body size. We analysed autopsy data to assess whether relative renal length and weight (e.g. corrected for body weight, height or body surface area (BSA)) are also larger in men. Assuming that kidney size is associated with nephron number, opposite findings could partly explain why women are less prone to the development and progression of chronic kidney disease than men.

**Methods:**

All forensic autopsies performed between 2009 and 2015 at the local university hospital of Geneva in individuals of European descent aged ≥18 years without a known history of diabetes and/or kidney disease were examined. Individuals with putrefied or severely injured bodies were excluded. Relative renal weight and length were respectively defined as renal weight divided by body weight or BSA and renal length divided by body height or BSA.

**Results:**

A total of 635 autopsies (68.7% men) were included in the analysis. Left kidneys were on average 8 g heavier and 2 mm longer than right kidneys (both: *p* < 0.05). Absolute renal weight (165 ± 40 vs 122 ± 29 g) and length (12.0 ± 1.3 vs 11.4 ± 1.1 cm) were higher in men. Relative renal weight was also higher in men, but relative renal length was larger in women. In multivariable regression analysis, body height, body weight, the degree of blood congestion or depletion at autopsy and age were determinants of renal weight, whereas arterial hypertension and smoking were not. Percentile curves of renal weight and length according to sex and body height were constructed.

**Conclusion:**

Absolute and relative renal weights were both smaller in women. This is in line with recent studies stating that nephron numbers are also lower in women. Relative renal length was longer in women, suggesting that female kidneys have a more elongated shape. In comparison with older autopsy studies, renal weight appears to be stable over time.

## Background

Chronic kidney disease (CKD) is a fast growing global public health problem affecting an estimated ~ 9–16% of the world population and ~ 10% in Switzerland [1]. The prevalence of arterial hypertension (AHT), by many considered as a disease of renal origin, is even higher around 30–45% [2]. There is an ongoing debate on the question whether women are less prone to develop and progress CKD than men. In this context, many (but not all) studies have reported a higher prevalence of CKD in men, especially before the age of 60 [3]. In a recent worldwide survey, the age-standardized prevalence of arterial hypertension in adults was also higher in men: 24% versus 20% in women [4].

In the general population, the decline of renal function -generally expressed as (estimated) glomerular filtration rate ((e)GFR)- appears to start earlier and is faster in healthy men as compared to women [5, 6]. Moreover, cohorts of CKD patients have shown that men with CKD progress faster to end stage renal disease (ESRD) than women [7], suggesting that intrinsic renal differences exist between men and women.

This debate is hampered by a lack of insight in the mechanisms underlying the presumed protective effect of gender. Experimental models suggest a protective effect of estrogens, but data in human remain controversial [3, 8]. A low nephron number (for example due to low birth weight) leads, according to the Brenner hypothesis, to a compensatory glomerular hypertrophy and hyperfiltration in the remaining functional glomeruli, and ultimately to glomerular sclerosis, hypertension and progressive renal failure [9]. As kidney size (weight or length) probably correlates with nephron number [10, 11], one may wonder if kidney size is larger in women than men, which would offer them protection against or during kidney disease.

It is well known that imaging studies performed in humans have merely reported the opposite, namely that kidney size is larger in men [12].

However, body size, expressed as weight and length, is an important factor to consider when analyzing sex differences in kidney size. In this context, relative kidney size (corrected for body weight or body surface area (BSA)) may offer more accurate information than absolute kidney size, and tell us if kidney size remains larger in men once corrected for body size. Only few reports have published data on relative kidney size or integrated body size in their analysis [12–14]. Besides, most studies used radiological imaging techniques to measure kidney dimensions instead of directly measuring renal dimensions, as is only possible at autopsy or kidney donation. One of the largest autopsy study to date reported a higher absolute renal weight and length in men, yet relative renal weight and length were not reported [15]. This study also did not integrate the degree of blood depletion. This parameter is clinically assessed by the pathologist, and provides clues to the underlying cause of death. For example, signs of blood depletion may be observed following deaths due to stab wounds, whereas renal stasis/congestion can be observed in case of acute intoxication (typically opioid intoxication) [16, 17]. The degree of blood depletion is also an important parameter when assessing kidney dimensions, as circulating and renal blood volume may theoretically influence renal weight.

The above cited autopsy study was performed in 2001, and based on data collected between 1987 and 1991. As the average body height and weight of the population are increasing, at least in the Occidental world [18], the reported reference values of kidney size and weight may be less accurate nowadays. For these reasons, recent autopsy data on sex-differences in absolute and relative size of kidneys would be welcome.

The main objective of this autopsy study was to assess whether there are sex-differences in absolute and relative kidney size in a modern Western population. We hypothesized that absolute kidney size (expressed as weight and length) would be larger in men, but that relative kidney size (corrected for body weight or BSA) would not differ. If in contrast relative kidney size appeared larger in women, this could partly explain why they are more protected against the development and progression of CKD than men. A second aim was to update reference values of renal weight and length by providing sex-specific centile curves.

## Methods

### Study population

This retrospective study was based on the medico-legal autopsies ordered by the Geneva authorities between 2009 and 2015, preserved in the archives of the University Center of Legal Medicine of Geneva and Lausanne (CURML).

Autopsies performed in adults of European descent (Caucasians) aged 18 and above whose deaths were sudden, unexpected or violent, or for which the cause of death was not considered as natural by the attending physician could be included in the analysis. Subjects who had known kidney disease or who suffered from conditions known to possibly affect kidney function (diabetes type 1 and 2) were excluded. Subjects were also excluded if the autopsy revealed unexpected kidney diseases such as polycystic disease, renal agenesis, glomerulonephritis or interstitial nephritis. Other exclusion criteria were: non-European descent, moderate to severe putrefaction, post-mortem interval more than 72 h between death / death discovery and autopsy, renal trauma and/or any condition capable of affecting the subject’s weight or height (e.g. body strongly mutilated by severe trauma, burned body and/or amputated body parts).

Subjects with missing information on renal weight were also excluded.

### Data collected

The following clinical data were collected: sex, age, cause of death, body weight and height, and presence of cardiovascular comorbidities (hypertension, myocardial infarction). Hypertension status was determined based on medical history and medication intake by the deceased. Smoking status, alcohol or drug abuse (history of or positive drug screening that includes cocaine, cannabis, morphine and other opiates) were also recorded. Toxicological screening was performed on clinical indication, at the discretion of the forensic specialist.

All subjects were categorized according to the presence of blood congestion or blood depletion. Three categories of congestion status were defined: congested status, normal amount of blood, or depleted status, at the discretion of the pathologist.

Autopsies were performed by different pathologists, including one of the authors, following the same techniques and protocol. At autopsy, the following macroscopic data were collected: weight and length of the left and the right kidney and heart weight. The weight of each kidney appears in all autopsy reports, according to the local protocol, whereas renal dimensions (height, width and thickness) were only measured in autopsies performed before 2011. All organ measurements were conducted using the same scale (Mettler Toledo SB16001), after removal of the renal capsule and perirenal fat. Measurements of renal length were carried out using a metric ruler (cm). Body height was measured from head to sole with a metric ruler while the corpse was lying on the autopsy table. The presence of renal cysts, renal scars and atherosclerosis (ATS) of the renal arteries was also registered.

### Statistical analysis

All variables were expressed as mean, median (min-max) or percentage, as appropriate. The Shapiro–Wilk test was used to assess whether data were normally distributed.

We defined the relative renal weight and the relative renal length in two ways. The relative renal weight was firstly computed as the ratio between renal weight and body weight and secondly by dividing renal weight by BSA. The BSA was computed according to the DuBois formula [19].

In the same way, the relative renal length was defined as the ratio between renal height and body height and by dividing renal height by BSA. The renal measures were compared with the Student’s t-test or Wilcoxon-Mann-Whitney test, according to the distribution of the variables.

Multiple linear regression analyses were applied to describe the relation between renal weight or length and different pre-defined independent parameters (sex, age, body height, body weight, smoking, known arterial hypertension and the degree of renal blood congestion). In case the conditions of linear regression were not satisfied, a log transformation was performed. The model with the smallest Akaike information criterion (AIC) and the best R^2^ value was kept in the final analysis. The direction of the relation between each independent variable and the response variable was expressed as the regression coefficient β and its 95% confidence interval. All analyses were performed using R (version 3.5.2).

The reference centile curves according to the gender and the renal laterality were defined by fitting a generalized additive model for location scale and shape (GAMLSS). The different regression models were tested with diverse distributions and cubic and penalized B-splines. The best model for each centile curve was selected based on the AICs. The one with the lowest AIC was chosen for each curve. The final models used penalized B-splines (P-splines) to smooth the curves. These analyses were performed with the GAMLSS package from R [20].

## Results

### Study population

The CURML in Geneva performed 1′166 forensic autopsies during the period 2009–2015. In total, 531 subjects were excluded from this study, leaving 635 subjects for the final analysis. The principal cause of exclusion was the presence of moderate to severe cadaverous alteration, responsible for 133 exclusions, followed by non-European descent (96 individuals). Fourteen cases with one or more missing kidneys were also excluded. Only one of these had unilateral agenesis (prevalence of 0.1%);10 persons were neurologically deceased donors who underwent bilateral kidney donation before the autopsy (see Supplementary Information, Table S1 for details).

Toxicological screening was performed in 361 of the included subjects (57.0%) and was positive in 83 persons (13.1%). The characteristics of all included subjects are shown in Table 1. The majority of subjects were men (68.7%). Men were on average 6 years younger than women. Almost half of the kidneys presented signs of congestions (45.5%) as compared to those with normal renal blood volume (38.9%) or a depletion status (15.6%).

**Table 1:** Clinical characteristics of the population. Data are expressed as mean (standard deviation), median (25-75th percentile) or percentage, as appropriate

	Participants (***n*** = 635)	Men (***n*** = 436)	Women (***n*** = 199)
**Age of death, years**	53.1 (17.6)	51.2 (17.0)	57.2 (19.0)
**Body Height, cm**	173.0 (9.9)	177.2 (7.9)	163.8 (7.3)
**Body Weight, kg**	75 (62–87)	80 (71–90)	61 (54–72)
**Smoking, number (%)**	162 (25.5)	125 (28.7)	37 (18.6)
**BMI, kg/m**^**2**^	24.7 (21.6–28.3)	25.4 (22.6–28.7)	23.1 (20.3–26.8)
**Body surface, m**^**2**^	1.9 (1.7–2.1)	2.0 (1.8–2.1)	1.7 (1.6–1.8)
**Blood degree, number (%)**			
Congested	289 (45.5)	217 (49.8)	72 (36.2)
No particularity	247 (38.9)	153 (35.1)	94 (47.2)
Depleted	99 (15.6)	66 (15.1)	33 (16.6)
**Cause of death, number (%)**			
Intoxication	103 (16.2)	60 (13.8)	43 (21.6)
Asphyxiation	83 (13.1)	62 (14.2)	21 (10.5)
Drowning	28 (4.4)	26 (5.1)	14 (7.0)
Cold weapon	15 (2.4)	15 (3.0)	3 (1.5)
Polytrauma	75 (11.8)	49 (11.2)	26 (13.1)
Gunshot	48 (7.6)	42 (9.6)	6 (3.0)
Other unnatural cause	81 (12.8)	57 (13.1)	24 (12.1)
Cardiac	89 (14.0)	72 (16.5)	17 (5.4)
Lung	21 (3.3)	12 (2.8)	9 (4.5)
Vascular	19 (3.0)	13 (3.0)	6 (3.0)
Other diverse natural cause	38 (6.0)	22 (5.3)	15 (7.5)
Natural but unknown origin	29 (4.6)	18 (4.1)	11 (5.5)
No information	6 (0.9)	2 (0.5)	4 (2.0)
**Arterial Hypertension**	88 (13.9)	61 (14.0)	27 (13.6)

### Absolute and relative renal measurements

Kidney dimensions are summarized in Table 2 A-B. In both men and women, the left kidney was significantly heavier than the right kidney. This was also true for the relative renal weight. In contrast, there was no difference in absolute and relative renal length between the right and left kidneys.

**Table 2:** Absolute and relative renal dimensions. The relative measurements were calculated according to the body weight, the body height and the body surface. A) Comparison of absolute and relative renal values according to kidney laterality. B) Comparison of absolute and relative renal data according to sex

	***n***	Left		***n***	Right		***P***-value
		***Mean (sd)***	***Median (25–75%)***		***Mean (sd)***	***Median (25–75%)***	
A/							
**Renal weight (g)**	635	155.8 (43.9)	150 (126–180)	635	147.6 (42.8)	140 (120–170)	< 0.001
**Renal weight /g) / body weight (kg)**	635	2.09 (0.55)	2.03 (1.73–2.41)	635	1.98 (0.49)	1.91 (1.60–2.28)	< 0.001
**Renal weight (g) / body surface (m**^**2**^**)**	631	82.1 (19.1)	79.8 (68.5–93.5)	631	77.6 (17.8)	75.7 (64.8–87.5)	< 0.001
**Renal length (cm)**	142	12.0 (1.2)	12 (11–13)	142	11.8 (1.3)	12 (11–12.5)	0.08
**Renal length (cm)/ body height (cm)**	142	0.068 (0.007)	0.068 (0.063–0.072)	142	0.069 (0.007)	0.069 (0.064–0.073)	0.9
**Renal length (cm) / body surface (m**^**2**^**)**	142	6.33 (0.79)	6.28 (5.8–6.9)	142	6.22 (0.80)	6.26 (5.54–6.70)	0.01
	***n***	**Men**		***n***	**Women**		***P*-value**
		***Mean (sd)***	***Median (25–75%)***		***Mean (sd)***	***Median (25–75%)***	
B/							
**Left renal weight (g)**	436	169.5 (42.0)	165 (140–195)	199	125.4 (31.2)	121 (103–141)	< 0.001
**Left renal weight (g) / body weight (kg)**	436	2.12 (0.51)	2.04 (1.75–2.41)	199	2.04 (0.64)	1.96 (1.59–2.37)	0.042
**Left renal weight (g) / body surface (m**^**2**^**)**	434	85.4 (18.6)	82.4 (72.0–96.8)	195	74.6 (17.9)	71.5 (63.4–84.7)	< 0.001
**Left renal length (cm)**	101	12.2 (1.3)	12 (11–13)	41	11.6 (1.1)	11 (10.5–12)	0.011
**Left renal length (cm)/ body height (cm)**	101	0.068 (0.007)	0.069 (0.063–0.072)	41	0.071 (0.007)	0.071 (0.065–0.075)	0.039
**Left renal length (cm) / body surface (m**^**2**^**)**	101	6.14 (0.69)	6.14 (5.66–6.55)	41	6.82 (0.81)	6.89 (6.15–7.35)	< 0.001
**Right renal weight (g)**	436	160.9 (41.4)	155 (132–182)	199	117.9 (28.7)	116 (97–133)	< 0.001
**Right renal weight (g) / body weight (kg)**	436	2.01 (0.46)	1.96 (1.63–2.29)	199	1.91 (0.54)	1.88 (1.49–2.25)	0.011
**Right renal weight (g) / body surface (m**^**2**^)	436	81.0 (17.6)	78.5 (68.4–90.3)	195	70.1 (15.9)	68.8 (58.2–79.0)	< 0.001
**Right renal length (cm)**	101	12.0 (1.3)	12 (11–12.5)	41	11.4 (1.1)	11 (10.5–12)	0.011
**Right renal length (cm) / body height (cm)**	101	0.067 (0.007)	0.066 (0.062–0.071)	41	0.069 (0.007)	0.069 (0.065–0.073)	0.03
**Right renal length (cm) / body surface (m**^**2**^**)**	101	6.02 (0.68)	6.11 (5.45–6.45)	41	6.69 (0.88)	6.61 (5.96–7.45)	< 0.001

**Fig. 1:**
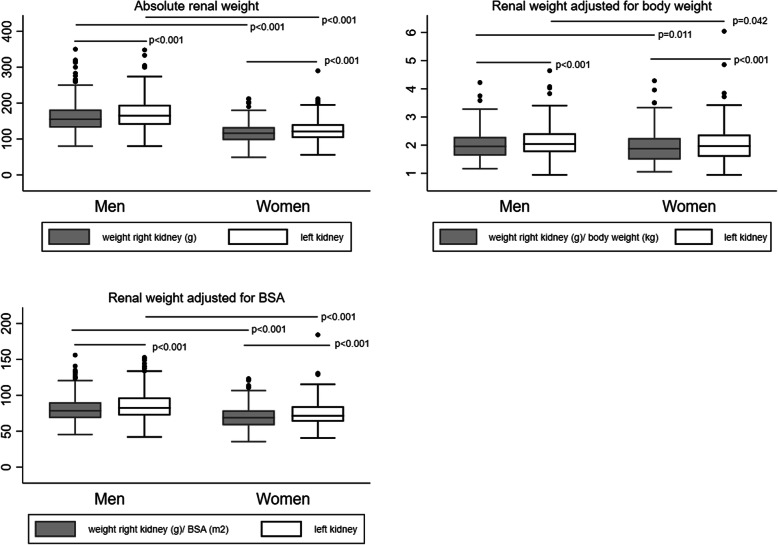
Absolute and relative renal weight according to gender. Left kidneys are heavier than right kidneys. Absolute and relative weight are higher in men

Absolute renal weight and length were higher in men for both kidneys. Relative weight was also higher in men (Fig. 1), but relative renal length was larger in women (Table 2B).

Important differences in renal weight were observed according to the congestion status. Depleted kidneys weighed on average 25.8 g (36–15.6 g) less than congested ones (*p* < 0.001). Kidneys with normal blood amount weighed 14.8 g (22.6–7.0 g) less than congested ones (*p* < 0.001).

### Determinants of renal weight

**Fig. 2:**
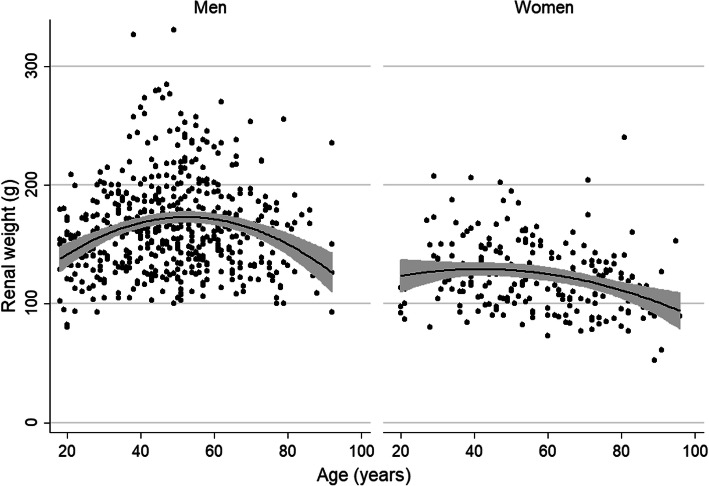
Age-related changes in renal weight according to gender

We applied a multiple linear regression to assess the associations between mean renal weight and the predefined variables sex, age, body height, body weight, smoking habit, known hypertension and renal blood degree. A log-transformation of renal weight was performed to fulfill the conditions of the regression. As shown in Table 3, a positive association was found between renal weight and body weight, body height and the degree of renal congestion. The analysis also demonstrated a negative association between renal weight, sex and the age at death. There was a difference between men and women: in men, renal weight slightly increased till the age of 52.5 years, and decreased sharply hereafter. In women, the relationship was flatter and decreased gradually after the age of 50 years (see Fig. 2). Renal weight was not associated with smoking habit or known arterial hypertension. The model included two interactions between the explanatory variables, respectively between body height and body weight (*P* < 0.011) and body height and age (*P* < 0.001).

**Table 3:** Multiple regression analysis showing associations between the outcome variable renal weight (log-transformed mean of both kidneys) and predefined clinical variables

	Estimate β	Standard error	***P***-value	
			unadjusted	adjusted
(Intercept)	4.641	0.0555	< 0.001	< 0.001
Blood degree (depletion)	−0.236	0.0718	< 0.001	0.001
Blood degree (no particularity)	−0.035	0.0571	< 0.001	0.54
Weight	0.0174	0.0046	< 0.001	< 0.001
Height	0.012	0.0004	0.024	< 0.001
Sex (women)	−0.149	0.022	< 0.001	< 0.001
Age of death	−0.0273	0.0059	0.035	< 0.001
Weight:height	−0.001	0.00003	< 0.001	0.011
Age of death:height	0.002	0.00003	0.51	< 0.001

### Determinants of renal length

A multiple regression analysis including the same explanatory variables as above was performed to assess which factors were associated with renal length in the 142 subjects with information on this parameter. Renal length was log-transformed. Renal length was not associated with the degree of blood congestion, smoking habit or the age of death. Significant positive associations were found with body weight, body height and sex for both kidneys (Table 4). Two significant interactions were identified: between body weight and height (*p* = 0.001) and between sex and body height (*p* = 0.01).

**Table 4:** Multiple regression analysis illustrating the associations between renal length (log-transformed mean of both kidneys) and clinical variables

	Estimate β	Standard error	***P***-value	
			unadjusted	adjusted
Sex (women)	0.198	0.065	0.009	0.003
Weight	0.045	0.0129	< 0.001	< 0.001
Height	0.021	0.0063	0.019	0.001
Age of death	0.0114	0.0082	0.27	0.17
Weight:height	−0.0024	0.0007	< 0.001	0.001
Sex (women):height	−0.0959	0.0366	0.29	0.01

### Centile curves

**Fig. 3:**
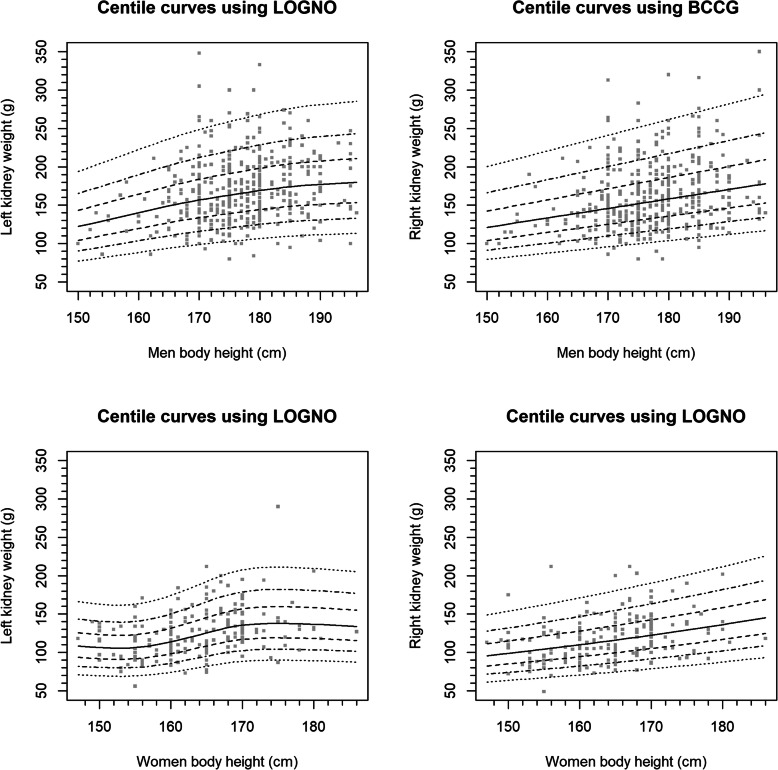
Centile curves of renal weight according to body height, gender and renal laterality. (LOGNO: log-Normal, BCCG: Box-Cox Cole and Green distribution)

The reference centile curves of renal weight according to sex, body height and laterality are shown in Fig. 3. Additional curves were created according to the degree of blood depletion (Supplementary information, Fig. S1 A-C).

## Discussion

The present study shows that absolute and relative renal weight (corrected for body weight and/or body surface area) are both greater in men than in women. This finding was not explained by differences in the prevalence of arterial hypertension or smoking habits. This result was also independent of the amount of blood in the kidneys (blood degree) at the time of the autopsy. As expected, blood-depleted kidneys were lighter and blood-congested kidneys heavier, but this did not affect the conclusion that the relative renal weight is greater in men than women. This autopsy study also shows that left kidneys are heavier (and thus larger) than right kidneys. Finally, absolute renal length was larger in men, but relative length (adjusted for body height) was larger in women, suggesting a more elongated shape of kidneys in women.

Our results show that gender is a determinant of renal weight, irrespective of anthropometric parameters. Hence, women have relatively smaller kidneys that are 25% lighter than men’s kidneys. This result is in line with a recent biopsy study in healthy kidney donors by Denic et al., who reported that male kidneys are not only larger in size, but also contain a higher number of nephrons [11]. Denic et al. estimated the nephron number indirectly by multiplying the glomerular density in protocol kidney biopsies with the cortical volume as measured by CT scan and a correction factor.

Why absolute and relative renal weights are larger in men cannot be answered by our retrospective autopsy study. High protein intake has according to some a positive impact on kidney size, and may therefore partly explain this difference [21]. Unfortunately, we have no data on dietary intake of proteins in the studied population, but we know that food and protein intake is higher in men in the Swiss population [22]. Hormonal factors also have a significant impact on kidney function and histology [23, 24]. Animal studies have shown that estradiol slows glomerulosclerosis and helps maintain kidney size in women [25]. In contrast, testosterone stimulates apoptosis of proximal tubular cells in animal studies [26]. If similar effects occur in humans, our study should have shown opposite results; as histological data and hormone levels were not available, we could not explore these sex hormone-kidney interactions.

In multivariable models, other determinants of renal weight were age, body height and body weight, whereas hypertension was not. Denic et al. found the same determinants in their study. Although these authors focused on nephron number, not renal weight, this is an interesting finding as it can be interpreted as an argument in favor of the notion that kidney size is a proxy of nephron number. However, this statement should be interpreted with caution, as the relationship between kidney size and nephron number may not be linear [27]. In both our study and the study by Denic et al., renal mass (expressed as nephron number or (relative) kidney size) was smaller in women [11]. Therefore, it seems reasonable to conclude that differences in renal mass do not explain the lower susceptibility of women to CKD and its progression.

Age was negatively associated with renal weight. Of interest, there was a difference between men and women: in men, renal weight slightly increased till the age of 52.5 years, and decreased sharply hereafter. In women, the relationship was flat and decreased gradually after the age of 50. These data are in line with a recent Italian population-based study in 2256 subjects that assessed kidney size with ultrasound [28]. The observed differences may be driven by sex hormones. For example, the initial increase in renal size in men may be caused by androgen-induced nephron hypertrophy [29]. The decrease in renal size may be caused by progressive nephrosclerosis, but we did not collect data on this variable. Microscopic studies are needed to further explore the association between age and renal size.

Left kidneys were on average 8 g heavier and 0.2 cm longer than right kidneys. This finding confirms the general paradigm that left kidneys are larger than right kidneys. Previous studies showed discrepant results, but were often limited by the indirect (radiological) methods used to measure kidney dimensions [13, 28]. It has been suggested that the perfusion and development of the right kidneys are hampered by the passage of the right renal artery posterior to the inferior vena cava and the larger volume taken by the liver as compared to the spleen, but this is hypothetical [30]. Whatever the reason, the small differences in weight and length are to our opinion clinically not relevant.

In our study, men’s kidneys were significantly longer than those of women. However, once adjusted to body weight and body surface area, renal length was higher in women. Cheong et al. observed the same results in 150 individuals with MRI-assessed kidneys length [31]. On the other hand, Miletić et al. found no significant differences between relative kidney length (adjusted for body size) of men and women using ultrasound [12]. This was also the result of a Swiss study that used ultrasound to evaluate renal weight and size in 793 healthy individuals [13]. These discrepancies may be once more due to the use of different radiological methods to estimate kidney dimensions. Our study was not limited by these technical factors since renal length was measured directly during the autopsy. Our results suggest that kidneys are shorter, heavier and thus thicker in men, and relatively longer, lighter and thinner in women. However, kidney length was the only dimension that was measured in this study; further studies that take the three kidney dimensions (length, width, and thickness) into account are needed to confirm this finding.

Our study provides updated sex-specific reference values for renal weight. The percentile curves may be of clinical use for pathologists and forensic specialists. For example, a renal weight under the 2.5th percentile may point towards CKD, whereas values over the 97.5th percentile may point towards underlying pathologies such as diabetes, amyloidosis and other storage diseases. The reference values are provided for both depleted, congested and normal kidneys, as renal weight varied according to blood degree. The percentile curves may also have some clinical implications in renal transplantation. Although today, one usually does not take into account kidney size to decide which kidney should be given to which patient, in general, larger donor kidneys result in better graft function at follow up [32]. The percentile curves in our article will allow clinicians to estimate expected kidney size of potential donors before organ removal or imaging.

Of note, results should be interpreted with caution, as there was a wide variance of renal weight at any renal size. This is possibly one of the reasons why no relationship was found between hypertension and renal weight in our study, nor between nephron number and blood pressure in recent large biopsy studies [27].

To the best of our knowledge, no update of reference values for the European population has been performed since the publication by de la Grandmaison et al. in 2001 [15]. As the average body height and length have increased over time under the influence of environmental and genetic factors [18], we believe that this update represents better the actual European population. To illustrate this, body height, weight and BMI were higher for men and women in our study compared to the study by de la Grandmaison et al. Of interest, renal weight in men was on average 3 g higher in our study, but 13.5 g lower in women. The younger age of women in the study by de la Grandmaison (49 ± 20 years vs 57 ± 19 years in our study) may partly explain this finding. Data from other, older studies show similar trends: over time a slightly higher renal weight for men and lower weight for women (see Table 5) [15, 17, 33]. However, the number of studies is too limited to draw definite conclusions and future studies are necessary to assess whether there is indeed a trend over time in women to lower kidney weight.

**Table 5:** Renal weight and body dimensions at autopsy in different studies over time. *M* Men, *W* Women, *R* right kidney, *L* left kidney

Study	Age (years)	Kidney Weight (g)		Body length (cm)		Body Mass Index (kg/m^**2**^)	
		*Men*	*Women*	*Men*	*Women*	*Men*	*Women*
Pourteyron, 1872		R = 141	R = 115				
		L = 152	L = 124				
De la Grandmaison, 2001	M = 42	R = 162	R = 135	172	161	22.8	22.5
	W = 49	L = 160	L = 136				
Molina, 2012	M = 24	R = 129	No women included	173		25.4	
		L = 137					
Molina, 2015	W = 24	No men included	R = 108		160		25.2
			L = 116				
Addidou Kalucki, 2020	M = 51	R = 161	R = 118	177	164	25.4	23.1
	W = 57	L = 170	L = 125				

Although this study is one of the largest ever performed in this field, it has some limitations. First of all, we do not have data on serum creatinine or other markers of renal function, and hypertension status was only based on medical records or drug intake. Therefore, our study may have included subjects whose kidney function was reduced because of age or associated diseases, and may have underestimated the number of hypertensives. Birth weight was also not available*.* Finally, substance abuse, especially cocaine, can alter renal function. A blood screening of substance abuse was performed on clinical indication, but a negative screening does not exclude previous use, nor the exposure to other potentially nephrotoxic medication such as non-steroidal anti-inflammatory drugs. At last, whether kidney dimensions corrected for height or BSA are more accurate in predicting the future of renal function remains to be demonstrated. Indeed, indexing other renal parameters such as glomerular filtration remains a matter of discussion and may induce errors in some clinical conditions [34].

## Conclusions

In summary, our study shows that renal weight (both absolute and relative) is larger in men than women, but the latter have more elongated kidneys. This study also provides new reference values for renal weight at renal autopsy, according to the degree of blood depletion.

Why men have relatively larger kidneys, and how kidney size correlates with nephron number needs further study, possibly with new imaging techniques such as micro-CT or cationized ferritin MRI that are capable of measuring directly the total number of nephrons in one kidney. So far, they are restricted to small animals, but this may change in the near future [35].

## Supplementary information

**Additional file 1.** Centile curves of renal weight taking into account depletion status of the kidneys.

## Data Availability

On reasonable request from the principal investigator (MP).

## Abbreviations

AHT: Arterial hypertension
BMI: Body mass index
BSA: Body surface area
CKD: Chronic kidney disease
eGFR: Estimated glomerular filtration rate
MRI: Magnetic resonance imaging
