# Supplementary material for: Reference values and sex differences in absolute and relative kidney size. A Swiss autopsy study
Source: BMC Nephrol. 2020 Jul 20;21:289. doi: 10.1186/s12882-020-01946-y (PMC7372852; doi:10.1186/s12882-020-01946-y)

**Supplementary material:**

**Table S1**: Exclusion criteria for the study subjects selection.

| **Subjects selection** | **Total** | **Men** | **Women** |
| --- | --- | --- | --- |
| Initial number of subjects | 1165 (100%) | 812 | 353 |
| Number of included subjects | 635 (54.5%) | 436 | 199 |
|  |  |  |  |
| Number of excluded subjects and reason for exclusion | 423 | 295 | 128 |
| *Putrefied corps* | 133 | 96 | 37 |
| *Known kidney disease* | 88 | 57 | 31 |
| *Diabetes mellitus* | 85 | 61 | 24 |
| *Age < 18 years old* | 40 | 21 | 19 |
| *Non-European descent* | 96 | 74 | 22 |
| *Renal trauma* | 38 | 26 | 12 |
| *Renal cyst > 5 cm* | 16 | 15 | 1 |
| *Absence of one or two kidneys* | 14 | 11 | 3 |
| *Others (e.g. charred body, amputation)* | 9 | 8 | 1 |

**Figure S1:** Centile curves of the A) depleted, B) with no particularity and C) congested renal weight in function of the body height according to the gender and the renal laterality over the body height. Several percentiles (2.5, 10, 25, 50, 75, 90, and 97.5) are represented for men and women without any conditions affecting kidney function or deterioration of the body. The centiles curves follow different distributions according the gender and the laterality (IG: Inverse Gaussian; RG: Reverse Gumbel; GA: Gamma, LOGNO: log-Normal).

A)


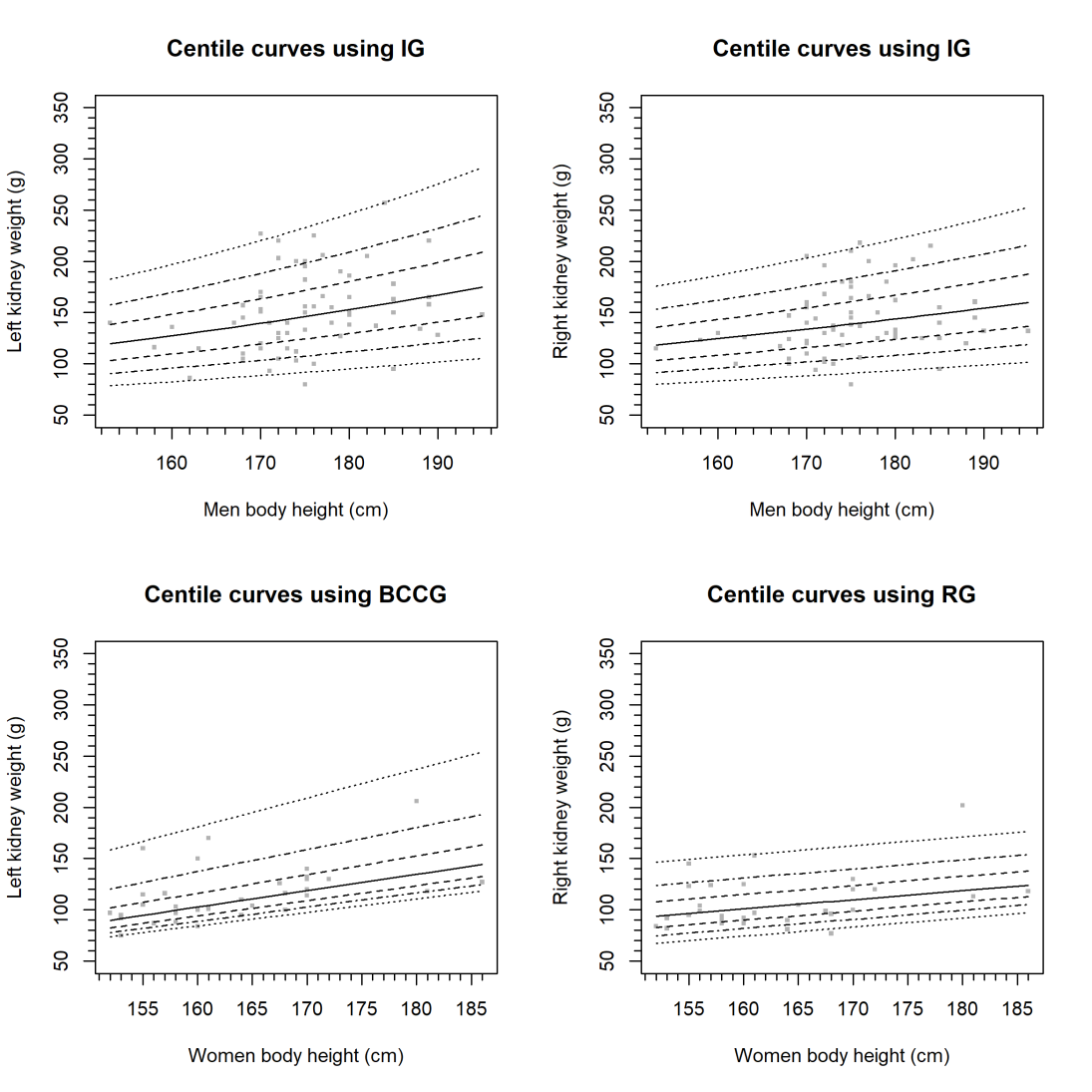


B)


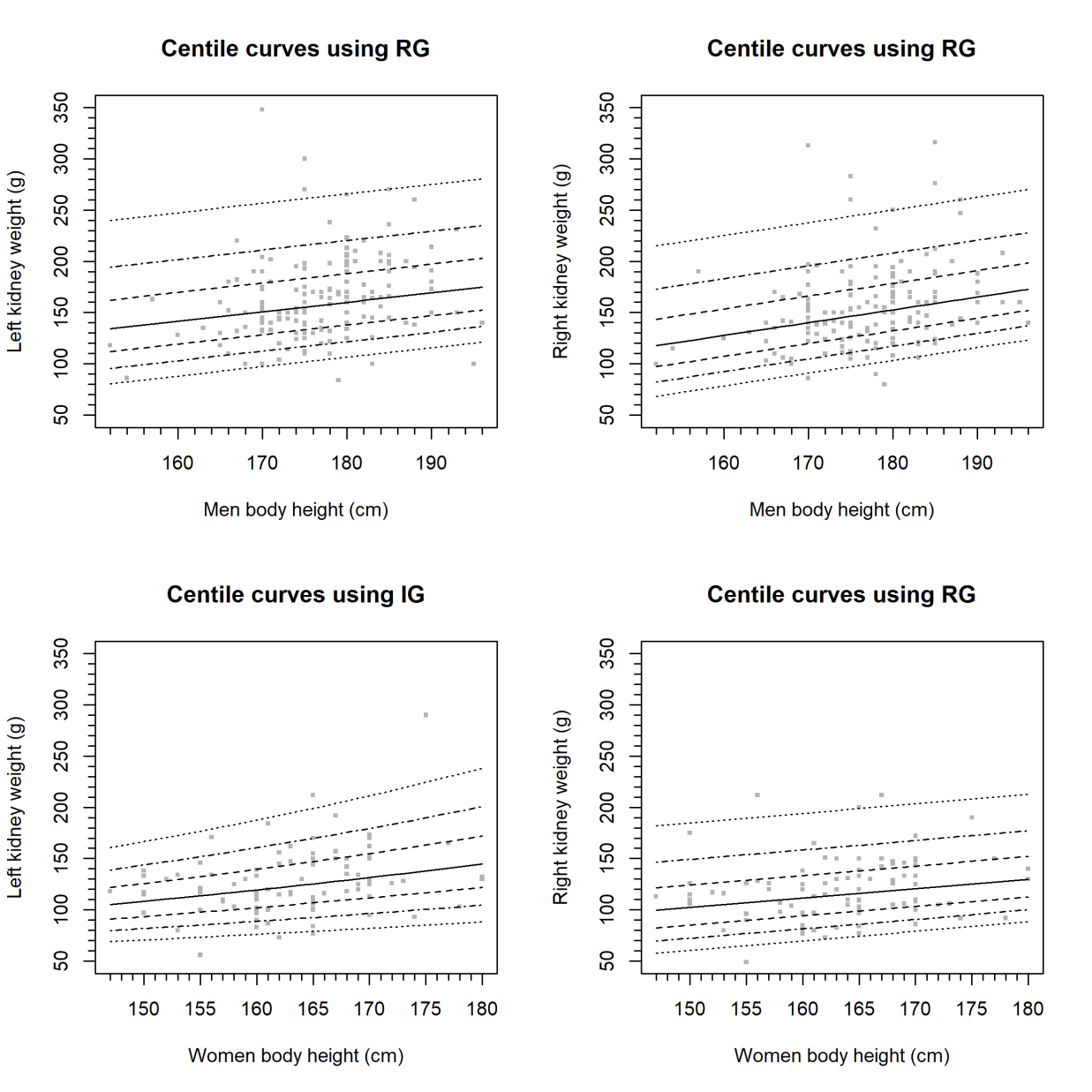


C)


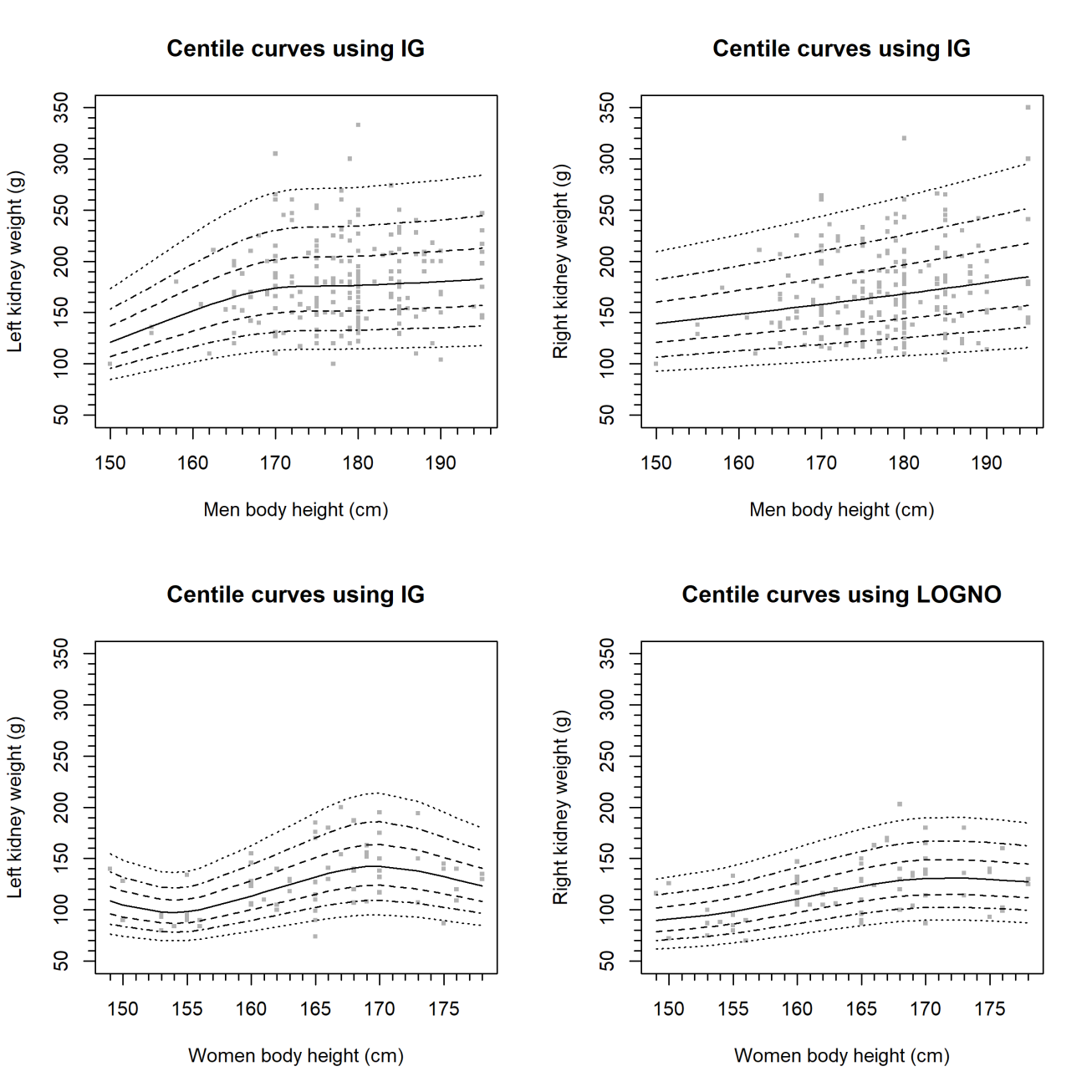

Supplement: Supplementary file 1 — Additional file 1. Centile curves of renal weight taking into account depletion status of the kidneys. [file 12882_2020_1946_MOESM1_ESM.docx]
